# An improved procedure for the preparation of Ru(bpz)_3_(PF_6_)_2_ via a high-yielding synthesis of 2,2’-bipyrazine

**DOI:** 10.3762/bjoc.11.9

**Published:** 2015-01-14

**Authors:** Danielle M Schultz, James W Sawicki, Tehshik P Yoon

**Affiliations:** 1 Department of Chemistry, University of Wisconsin–Madison, 1101 University Avenue, Madison, WI 53706, USA

**Keywords:** heterocycles, ligand synthesis, palladium, photocatalysis, reductive coupling

## Abstract

There has been a recent surge of interest in the use of transition metal polypyridyl complexes as visible light-absorbing photocatalysts for synthetic applications. Among the most attractive features of this approach is the availability of many known complexes with well-characterized photophysical and electrochemical properties. In particular, Ru(bpz)_3_^2+^ is a powerful photooxidant that has proven to be uniquely suited for oxidatively induced photoredox transformations. We present here a straightforward and high-yielding route to Ru(bpz)_3_(PF_6_)_2_ that features an improved Pd-catalyzed synthesis of the 2,2’-bipyrazine ligand that is amenable to gram-scale preparations.

## Findings

Visible light-photoredox catalysis using transition metal chromophores is rapidly becoming recognized as an important strategy in organic synthesis [1–5]. This approach towards reaction design enables the facile generation and exploitation of odd-electron intermediates such as radicals and radical ions under exceptionally mild reaction conditions. A large number of transition metal chromophores with well-characterized photophysical and electrochemical properties are known, and the influence of ligand modification on the photoredox properties of these complexes is well understood [6–8]. As a result, a variety of Ru and Ir based chromophores spanning a range of redox potentials have recently become widely utilized in the design of new photocatalytic transformations [9].

**Figure 1:**
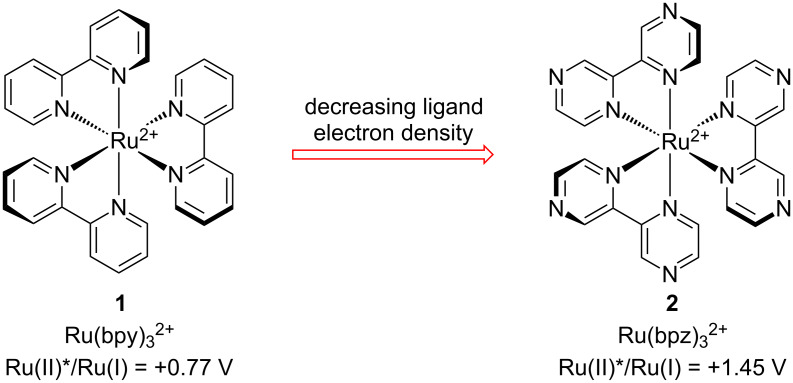
Influence of ligand structure on photoredox behavior.

In particular, the homoleptic tris(bipyrazyl) complex **2** (Ru(bpz)_3_^2+^) [10] has emerged as one of the most useful transition metal photocatalysts for oxidatively induced organic transformations (Figure 1). Due to the electron-deficient nature of its bipyrazyl ligands, the excited state redox potential of **2** is quite positive (+1.45 V vs SCE) [11]. Consequently, it is an effective photocatalyst in oxidatively induced photoredox transformations where less strongly oxidizing complexes (e.g., **1**) are not successful. For instance, we have reported that **2** is uniquely capable of promoting radical cation mediated Diels–Alder cycloadditions [12], radical thiol–ene couplings [13–14], and photooxygenation reactions [15–16]. Similarly, Zheng has reported oxidatively initiated indole synthesis [17] and [3 + 2] cycloaddition [18–19] reactions using photocatalyst **2**. Finally, a variety of transition metal complexes bearing bipyrazyl ligands have been prepared and investigated for a wide range of applications in inorganic and organometallic chemistry [20–25].

**Scheme 1:**
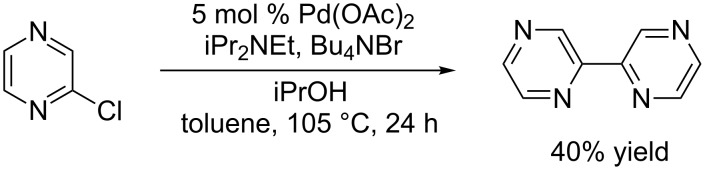
Pd-catalyzed reductive coupling of 2-chloropyrazine.

Unfortunately, the synthesis of 2,2’-bipyrazyl (bpz) is quite challenging compared to the syntheses of other, structurally similar bidentate bisazene ligands. Various methods for the preparation of bpz have been reported [26–28], the most common of which involve transition metal-catalyzed reductive homocouplings of halopyrazine electrophiles [29–30]. However, we found these procedures to be capricious in our hands, and after a survey of known reductive dimerization protocols, the highest yields of bpz we were able to obtain resulted from a Pd-catalyzed procedure reported by Plé (Scheme 1) [31]. Using this protocol, we were able to obtain only 40% yield of the desired 2,2-bipyrazine ligand on milligram scale, accompanied by a significant degree of undesired reductive dehalogenation. Moreover, the yields on larger scales were unreliable, and work-up and purification of this inefficient coupling reaction proved difficult.

Thus, in order to accelerate our investigations of complex **2** as a strongly oxidizing photoredox catalyst, we required a robust, high-yielding, and scalable synthesis of bpz. An improved synthesis of this ligand would be useful both in the context of the growing interest in photoredox catalysis as well as other organometallic and inorganic applications of bpz-supported transition metal complexes.

Our optimization studies for the reductive coupling of 2-halopyrazines are summarized in Table 1. Initial exploratory experiments using 2-chloropyrazine as the substrate gave quite poor yields using either organic or inorganic bases (Table 1, entries 1 and 2). Speculating that oxidative addition into the aryl chloride bond might be problematic in this coupling reaction, we turned our attention towards the use of more reactive aryl iodides. A screen of solvents commonly used in Pd-catalyzed cross-coupling reactions revealed that DMF provided the highest yields (Table 1, entries 3–5). We next examined the effect of varying equivalents of the terminal reductant, and we observed that the presence of excess isopropanol had little effect (Table 1, entries 6 and 7). Finally, an evaluation of the reaction temperature revealed that the coupling proceeded sluggishly at lower temperatures (Table 1, entry 8). Optimal conditions thus called for 5 mol % Pd(OAc)_2_, 2 equiv of isopropanol, and 1.5 equiv of K_2_CO_3_ in DMF (0.4 M) at 100 °C; under these conditions, the reaction was complete in 2 h and afforded the desired homocoupling product in 81% isolated yield (Table 1, entry 9).

**Table 1:** Optimization of Pd-catalyzed reductive homocoupling of 2-halopyrazines.^a^

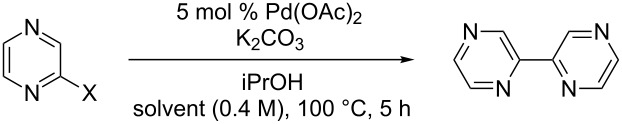				

entry	X	solvent	equiv iPrOH	yield^b^

1^c^	Cl	toluene	3	3%
2	Cl	toluene	3	3%
3	I	toluene	3	4%
4	I	dioxane	3	10%
5	I	DMF	3	85%
6	I	DMF	2	87%
7	I	DMF	1	83%
8^d^	I	DMF	2	44%
**9^e^**	**I**	**DMF**	**2**	**85% (81%)^f^**

^a^Conditions: Reactions were conducted on a 0.22 mmol scale using 1 equiv 2-halopyrazine, 5 mol % Pd(OAc)_2_, and 1.5 equiv K_2_CO_3_ at 100 °C unless otherwise noted. ^b^Yields determined by ^1^H NMR analysis using an internal standard. ^c^Reaction conducted using 1.5 equiv iPr_2_NEt instead of K_2_CO_3_. ^d^Reaction conducted at 60 °C. ^e^Reaction conducted for 2 h. ^f^Value in parentheses is the average isolated yield from two reproduced experiments.

Although our investigations were motivated by our specific need to access photocatalyst complex **2**, we were pleased to find that the optimal conditions for the synthesis of 2,2’-bipyrazine translated smoothly to the reductive coupling of a variety of pyridyl electrophiles. Table 2 demonstrates the application of these reaction conditions to the syntheses of a number of electron-deficient bipyridyl ligands that have been used to prepare strongly photooxidizing metal–polypyridyl complexes [32–36]. In each case, the requisite aryl iodides are either commercially available or can be easily synthesized by Finkelstein displacement of the aryl chloride with NaI [37].

**Table 2:** Pd-Catalyzed synthesis of other ligands used to support electron-deficient Ru(II) chromophores.^a^

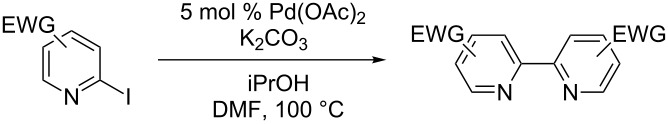				

entry	iodoarene	product	time	yield^b^

1	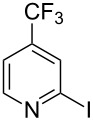	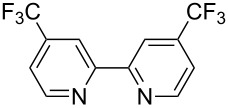	4 h	89%
2	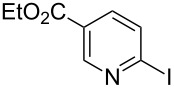	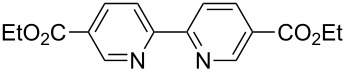	6 h	85%
3	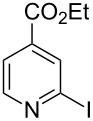	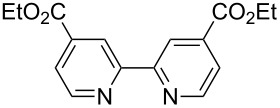	5 h	88%

^a^Conditions: 1.0 equiv 2-iodopyrazine, 1.5 equiv K_2_CO_3_, 2 equiv isopropanol, 5% Pd(OAc)_2_, DMF (0.4 M), 100 °C. ^b^Isolated yield (average of two experiments).

**Scheme 2:**
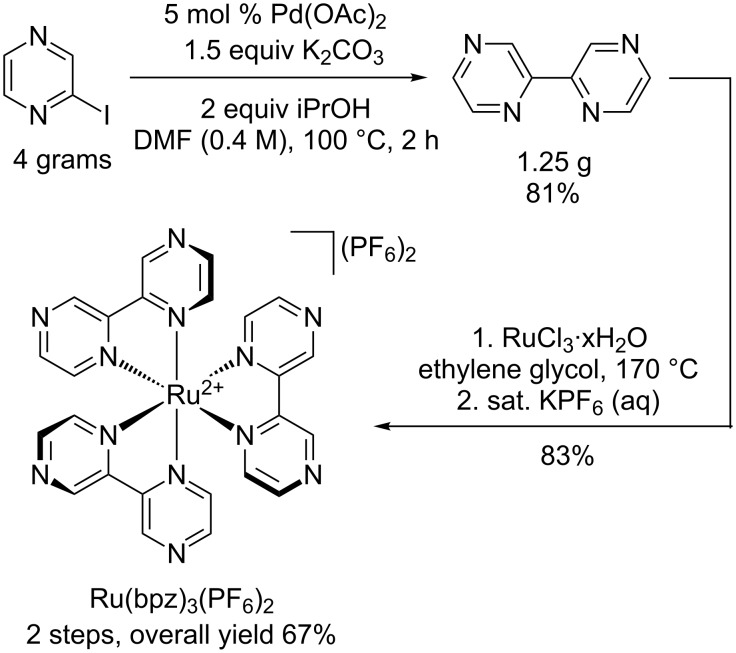
Preparative-scale synthesis of Ru(bpz)_3_(PF_6_)_2_.

Finally, we explored the scalability of the optimized conditions, and found that they remained applicable on multi-gram scale (Scheme 2). Thus, exposure of 4 g of 2-iodopyrazine to the optimized conditions from Table 1 cleanly produced 1.25 g of 2,2’-bipyrazine in 81% isolated yield. A portion of this material was then carried on to synthesize 689 mg (83% yield) of Ru(bpz)_3_PF_6_, showing for the first time that we could efficiently prepare hundreds of milligrams of this important photocatalyst. Importantly, this protocol has proven to be reproducible in the hands of other researchers as well [38].

In conclusion, we have developed an improved, Pd-catalyzed method for the synthesis of 2,2’-bipyrazine, an important ligand with growing utility in the context of visible light photocatalysis. This method is readily scalable to enable the gram-scale preparation of 2,2’-bipryazine, which facilitates the preparative synthesis of the strongly oxidizing photocatalyst Ru(bpz)_3_(PF_6_)_2_ [39].

## Supporting Information

File 1 Experimental section.
