# Supplementary material for: An improved procedure for the preparation of Ru(bpz)3(PF6)2 via a high-yielding synthesis of 2,2’-bipyrazine
Source: Beilstein J Org Chem. 2015 Jan 14;11:61–5. doi: 10.3762/bjoc.11.9 (PMC4311765; doi:10.3762/bjoc.11.9)
Supplement: File 1 — Experimental section. [file Beilstein_J_Org_Chem-11-61-s001.pdf]

Supporting Information

for

An improved procedure for the preparation of $Ru(bpz)_3(PF_6)_2$ via a high-yielding synthesis of 2,2'-bipyrazine

Danielle M. Schultz, James W. Sawicki and Tehshik P. Yoon*

Address: Department of Chemistry, University of Wisconsin–Madison, 1101 University Avenue, Madison, WI 53706, USA

*Corresponding author

Email: Tehshik P. Yoon – tyoon@chem.wisc.edu

Experimental section

General information. All reactions were performed open to air using standard glassware. All reagents were purchased from commercial sources and used without further purification. NMR data were obtained using 400 or 500 MHz spectrometers and are referenced to TMS. The NMR facilities at UW-Madison are supported by the NSF (CHE-1048642, CHE-9208463, S10 RR08389-01), the University of Wisconsin, and a generous gift from Paul J. Bender. IR spectral data were obtained using a ATR spectrometer (neat). Melting points are uncorrected.

Ethyl 2-iodoisonicotinate. A 100 mL round-bottomed flask was charged with ethyl 2-chloroisonicotinate (3.19 g, 17.2 mmol), NaI (6.18 g, 41.2 mmol), AcOH (1.96 mL, 34.4 mmol) and 20 mL of MeCN. To this was then added H_2SO_4 (91 μL, 1.72 mmol), which resulted in the reaction mixture turning bright red. The reaction was equipped with a water-cooled condenser and placed in a 90 °C oil bath for 18 h. The reaction was then cooled to room temperature and the solvent was removed *in vacuo*. To the remaining residue was added 50 mL of H_2O and solid $NaHCO_3$ until the reaction mixture was pH 7. The aqueous mixture was then transferred to a separatory funnel and extracted with CH_2Cl_2 (3 × 50 mL). Residual iodine was removed by washing the combined organic layers with a saturated solution of sodium bisulfite (50 mL). The organic layer was washed with brine (1 × 50 mL), dried with Na_2SO_4, and solvent was removed *in vacuo* to afford a yellow oil that was purified via distillation under reduced pressure to yield 3.23 g (68%) of the desired product as a white solid. ^1H NMR (500 MHz, $CDCl_3$) δ 8.52 (d, J =

4.9 Hz, 1H), 8.27 (s, 1H), 7.81 (dd, *J* = 5.0, 1.4 Hz, 1H), 4.42 (q, *J* = 7.1 Hz, 2H), 1.41 (t, *J* = 7.1 Hz, 3H). ^{13}C NMR (126 MHz, $CDCl_3$) δ 163.5, 151.3, 139.0, 134.2, 122.2, 118.1, 62.2, 14.2. IR (neat) 2986, 1724, 1541, 1382, 1250, 1137, 756 cm^{-1}. mp = 37 °C. HRMS (ESI) calculated for $[C_8H_8INO_2 + H]^+$ requires *m/z* 277.9673, found 277.9686.

General procedure for the Pd-catalyzed reductive homocoupling of 2-iodopyrazine and 2-iodopyridines for biaryl ligand synthesis: A 100 mL round bottom flask was equipped with a magnetic stir bar and charged with the appropriate aryl iodide (1 equiv), K_2CO_3 (1.5 equiv), iPrOH (2 equiv), $Pd(OAc)_2$ (5 mol %), and a sufficient volume of DMF to make a 0.35 M solution. The reaction mixture was placed under nitrogen and heated to 100 °C for 2–5 h until the starting material was consumed as judged by TLC analysis. The reaction mixture was cooled to room temperature and brine (40 mL) was added. The resulting mixture was transferred to a separatory funnel and extracted with EtOAc (3 × 75 mL). The organic layers were combined, washed with brine (1 × 75 mL), dried with Na_2SO_4, and the solvent was removed *in vacuo* to afford the crude product as a solid. The crude solid was purified by flash column chromatography or by recrystallization.

2,2'-Bipyrazine (Table 1, entry 9). Experiment 1: Prepared according to the general procedure using 4.02 g (19.4 mmol) 2-iodopyrazine, 4.02 g (29.1 mmol) K_2CO_3, 3.0 mL (38.8 mmol) iPrOH, 217 mg $Pd(OAc)_2$ (0.996 mmol), 55 mL DMF, and a reaction time of 2 h. The crude product was purified by flash column chromatography on silica gel to afford 1.25 g (81%) of 2,2'-bipyrazine as a beige solid. Alternatively, purification can also be achieved via recrystallization of the crude product from EtOAc/hexanes. Experiment 2: 3.06 g (14.9 mmol) 2-iodopyrazine, 3.08 g (22.3 mmol) K_2CO_3, 2.29 mL (29.8 mmol) iPrOH, 167 mg $Pd(OAc)_2$ (0.745 mmol), and 40 mL DMF. Isolated 960 mg (81%) of the desired product. ^{1}H NMR (400 MHz, $CDCl_3$) δ 9.61 (s, 2H), 8.67 (s, 4H). ^{13}C NMR (100 MHz, $CDCl_3$) δ 149.3, 145.3, 143.8, 143.5. IR (neat) 3076, 3013, 1523, 1382, 1088, 1027, 1016, 845 cm^{-1}. mp = 186–187 °C. These data are consistent with previously reported values [1].

4,4'-Bis(trifluoromethyl)-2,2'-bipyridine (Table 2, entry 1). Experiment 1: Prepared according to the general procedure using 3.01 g (11.0 mmol) 2-iodo-4-(trifluoromethyl)pyridine, 2.28 g (16.5 mmol) K_2CO_3, 1.70 mL (22.0 mmol) iPrOH, 123 mg $Pd(OAc)_2$ (0.548 mmol), 30 mL DMF, and a reaction time of 5 h. The crude product was purified by flash column chromatography on silica gel to afford 1.44 g (89%) of 4,4'-bis(trifluoromethyl)-2,2'-bipyridine as an off-white solid. Experiment 2: 2.0 g (7.33 mmol) 2-iodo-4-(trifluoromethyl)pyridine, 1.52 g (10.9 mmol) K_2CO_3, 1.13 mL (14.7 mmol) iPrOH, 82.2 mg $Pd(OAc)_2$ (0.366 mmol), and 20 mL DMF. Isolated 955 mg (89%) of the desired product. ^{1}H NMR (400 MHz, $CDCl_3$) δ 8.89 (d, *J* = 5.0 Hz, 2H), 8.73 (s, 2H), 7.59 (d, *J* = 5.0 Hz, 2H), ^{13}C NMR (125 MHz, $CDCl_3$) δ 156.1, 150.3, 139.6 (q, *J* = 34.2 Hz), 122.8 (q, *J* = 273.3 Hz), 119.9 (q, *J* = 3.5 Hz), 117.2 (q, *J* = 3.7 Hz). IR (neat) 3078, 1606, 1569, 1374, 1317, 1122, 668 cm^{-1}. mp = 78–79 °C. These data are consistent with previously reported values [2].

Diethyl [2,2'-bipyridine]-5,5'-dicarboxylate (Table 2, entry 2). Experiment 1: Prepared according to the general procedure using 2.05 g (7.40 mmol) ethyl 6-iodonicotinate, 1.50 g (10.8

mmol) K_2CO_3, 1.11 mL (14.4 mmol) iPrOH, 83.0 mg $Pd(OAc)_2$ (0.370 mmol), 20 mL DMF, and a reaction time of 6 h. The crude product was purified by flash column chromatography on silica gel to afford 933 mg (84%) of diethyl [2,2'-bipyridine]-5,5'-dicarboxylate as a white solid. Experiment 2: 1.50 g (5.41 mmol) ethyl 6-iodonicotinate, 1.15 g (8.33 mmol) K_2CO_3, 0.856 mL (11.1 mmol) iPrOH, 62.4 mg $Pd(OAc)_2$ (0.277 mmol), and 15 mL DMF. Isolated 701 mg (86%) of the desired product. ^{1}H NMR (400 MHz, $CDCl_3$) δ 9.30 (d, *J* = 2.0 Hz, 2H), 8.58 (d, *J* = 8.0 Hz, 2H), 8.44 (dd, *J* = 2.4, 8.4 Hz, 2H), 4.45 (q, *J* = 7.2 Hz, 4H), 1.44 (t, *J* = 7.2 Hz, 6H). ^{13}C NMR (125 MHz, $CDCl_3$) δ 165.2, 158.3, 150.6, 138.1, 126.6, 121.3, 61.5, 14.3. IR (neat) 2984, 1713, 1590, 1264, 1108, 1020, 760 cm^{-1}. mp = 146–147 °C. These data are consistent with previously reported values [3].

Diethyl [2,2'-bipyridine]-4,4'-dicarboxylate (Table 2, entry 3). Experiment 1: Prepared according to the general procedure using 1.00 g (3.61 mmol) ethyl 2-iodoisonicotinate, 748 mg (5.41 mmol) K_2CO_3, 0.556 mL (7.22 mmol) iPrOH, 40.5 mg $Pd(OAc)_2$ (0.180 mmol), 10 mL DMF, and a reaction time of 5 h. The crude product was purified by flash column chromatography on silica gel to afford 477 mg (88%) of diethyl [2,2'-bipyridine]-4,4'-dicarboxylate as a tan solid. Alternatively, purification can also be achieved via recrystallization of the crude product from hot EtOH. Experiment 2: 1.00 g (3.61 mmol) ethyl 2-iodoisonicotinate, 748 mg (5.41 mmol) K_2CO_3, 0.556 mL (7.22 mmol) iPrOH, 40.5 mg $Pd(OAc)_2$ (0.180 mmol), and 10 mL DMF. Isolated 479 mg (88%) of the desired product. ^{1}H NMR (500 MHz, $CDCl_3$) δ 8.95 (s, 2H), 8.87 (d, *J* = 4.9 Hz, 2H), 7.92 (dd, *J* = 4.9, 1.5 Hz, 2H), 4.47 (q, *J* = 7.1 Hz, 4H), 1.45 (t, *J* = 7.1 Hz, 6H). ^{13}C NMR (126 MHz, $CDCl_3$) δ 165.2, 156.5, 150.1, 139.0, 123.2, 120.5, 61.9, 14.3. IR (neat) 2986, 1721, 1594, 1250, 1104, 757 cm^{-1}. mp = 157 °C. These data are consistent with previously reported values [4].

Preparation of $Ru(bpz)_3(PF_6)_2$. Following a procedure adapted by Conrad and coworkers[5], a 50 mL round bottom flask was charged with $RuCl_3{\cdot}xH_2O$ (200 mg, 0.960 mmol), 2,2'-bipyrazine (632 mg, 3.99 mmol), and ethylene glycol (20 mL, 0.05 M) before being equipped with a water cooled condenser and heated to 170 °C for 16 h under an inert atmosphere. After the allotted time had elapsed, the reaction mixture was cooled to room temperature and saturated aqueous KPF_6 (9 mL) was added and allowed to stir for 2 h. The rusty red reaction mixture was filtered through a medium frit and washed with H_2O several times before equipping a clean collection flask and eluting the crude product with MeCN. Solvent removal *in vacuo* afforded a rusty brown solid that was purified by gravity column chromatography on alumina, eluting with MeCN. The red-colored fractions that eluted first were pooled, concentrated, and dried to afford 689 mg (83%) of $Ru(bpz)_3(PF_6)_2$ as an orange solid. ^{1}H NMR (500 MHz, DMSO-d_6) δ 10.12 (d, *J* = 1.3 Hz, 3H), 8.71 (d, *J* = 3.3 Hz, 3H), 8.00 (dd, *J* = 3.3, 1.2 Hz, 3H). ^{13}C NMR (126 MHz, DMSO-d_6) δ 150.3, 148.5, 147.0, 145.4. ^{19}F NMR (377 MHz, DMSO-d_6) δ –70.13 (d, J = 711.2 Hz). IR (neat) 3104, 1588, 1409, 821, 750 cm^{-1}. These data are consistent with previously reported values [6].

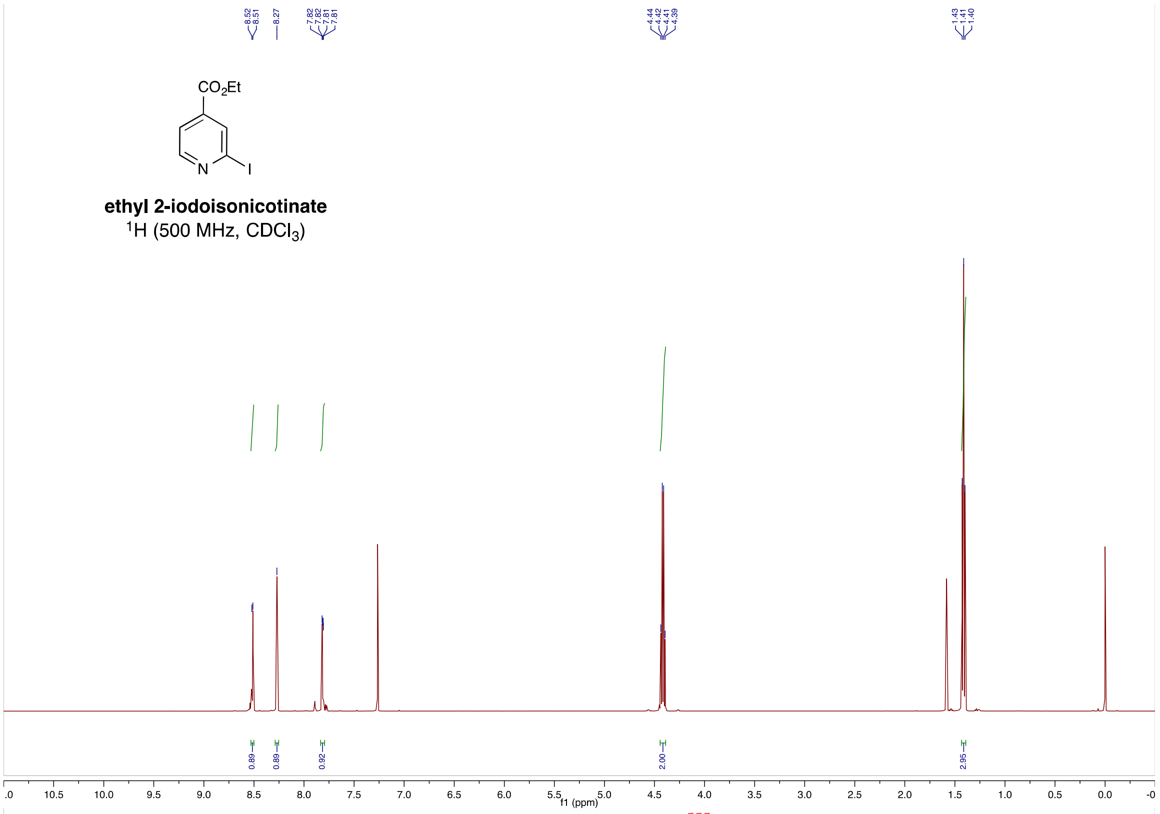
8.52
8.51
8.27
7.82
7.82
7.81
7.81
4.44
4.42
4.41
4.39
1.43
1.41
1.40
CO2Et
N
I
ethyl 2-iodoisonicotinate
1H (500 MHz, CDCl3)
0.89
0.89
0.92
2.00
2.95
f1 (ppm)

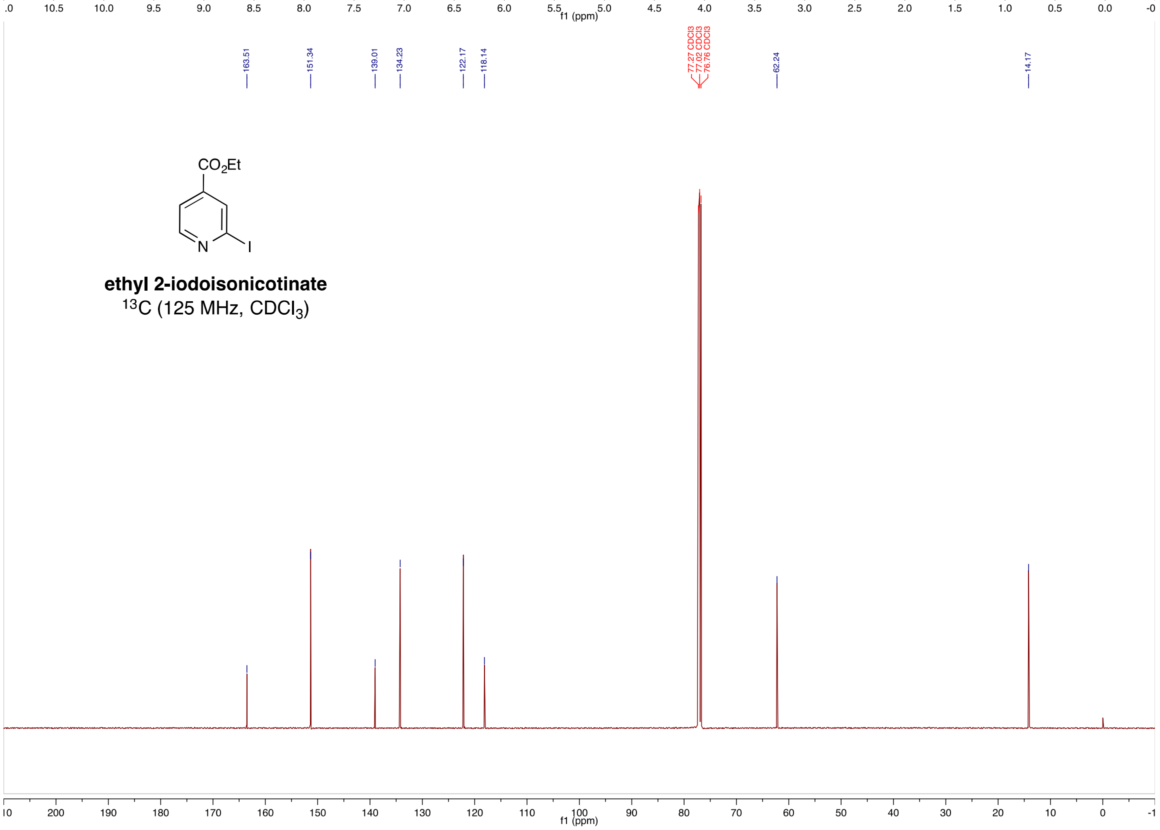
163.51
151.34
139.01
134.23
122.17
118.14
77.27 CDCl3
77.02 CDCl3
76.76 CDCl3
62.24
14.17
CO2Et
N
I
ethyl 2-iodoisonicotinate
13C (125 MHz, CDCl3)
f1 (ppm)

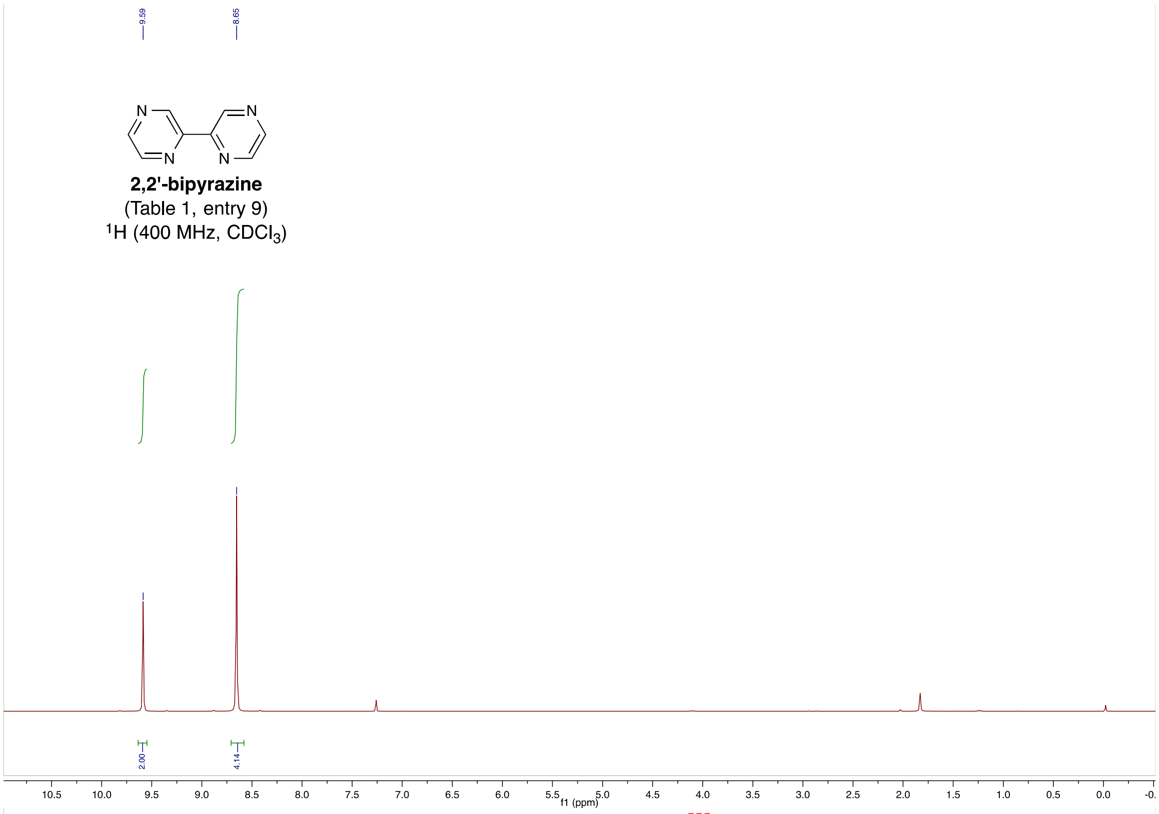
9.59
8.65
2,2'-bipyrazine
(Table 1, entry 9)
^{1}H (400 MHz, $CDCl_3$)
2.00
4.14
f1 (ppm)

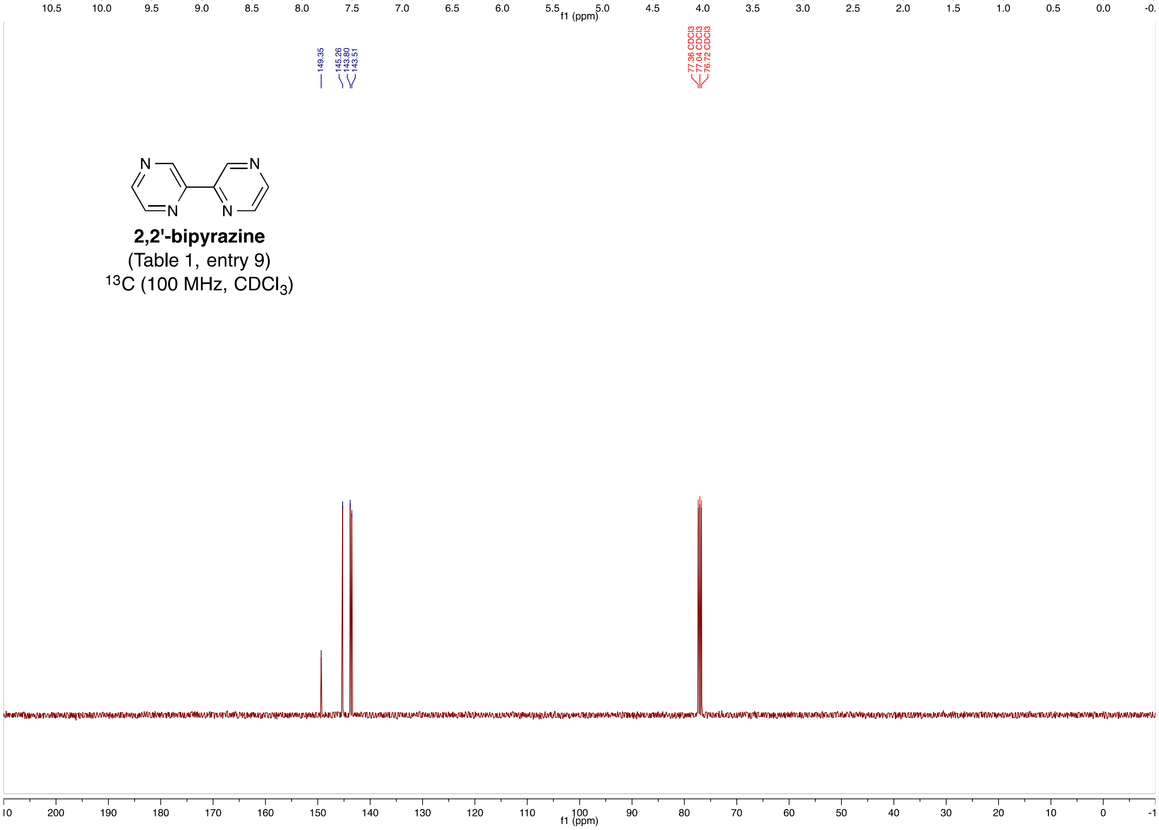
149.35
145.26
143.80
143.51
77.36 CDCl3
77.04 CDCl3
76.72 CDCl3
2,2'-bipyrazine
(Table 1, entry 9)
^{13}C (100 MHz, $CDCl_3$)
f1 (ppm)

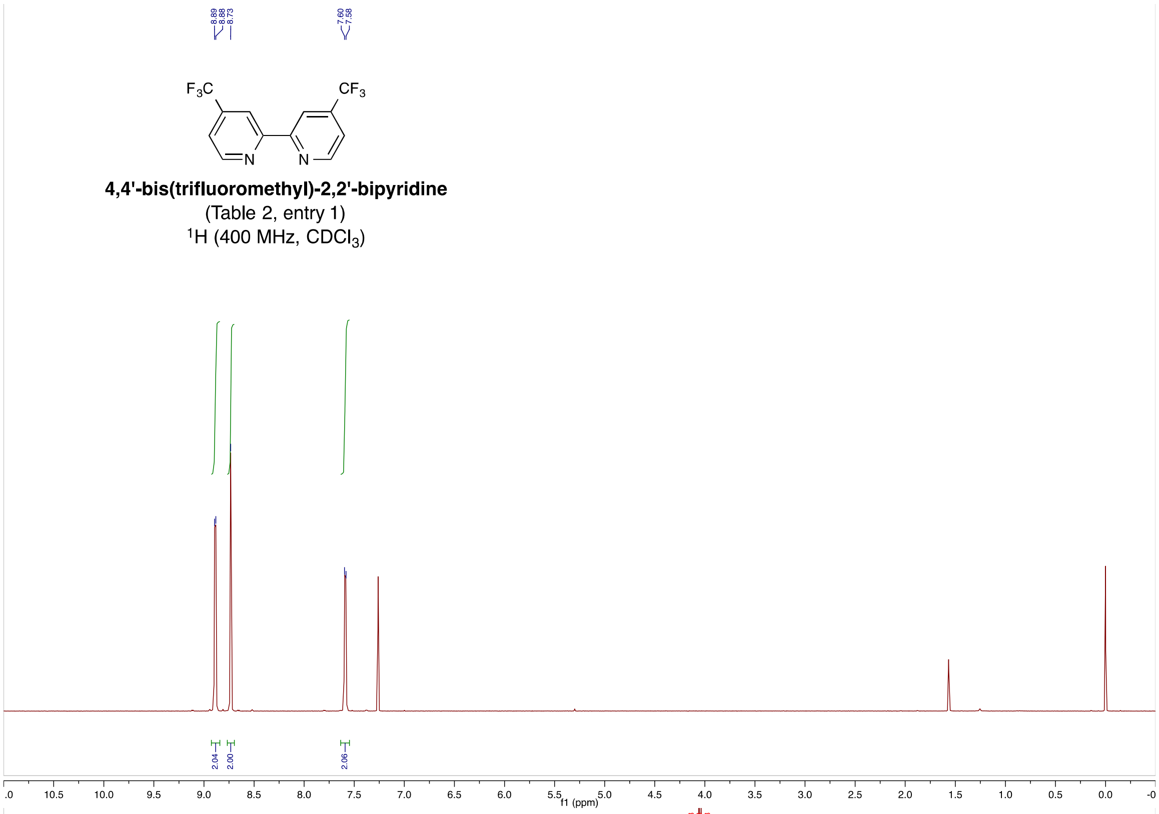
8.89
8.88
8.73
7.60
7.58
F3C
CF3
N
N
4,4'-bis(trifluoromethyl)-2,2'-bipyridine
(Table 2, entry 1)
1H (400 MHz, CDCl3)
2.04
2.00
2.06
f1 (ppm)

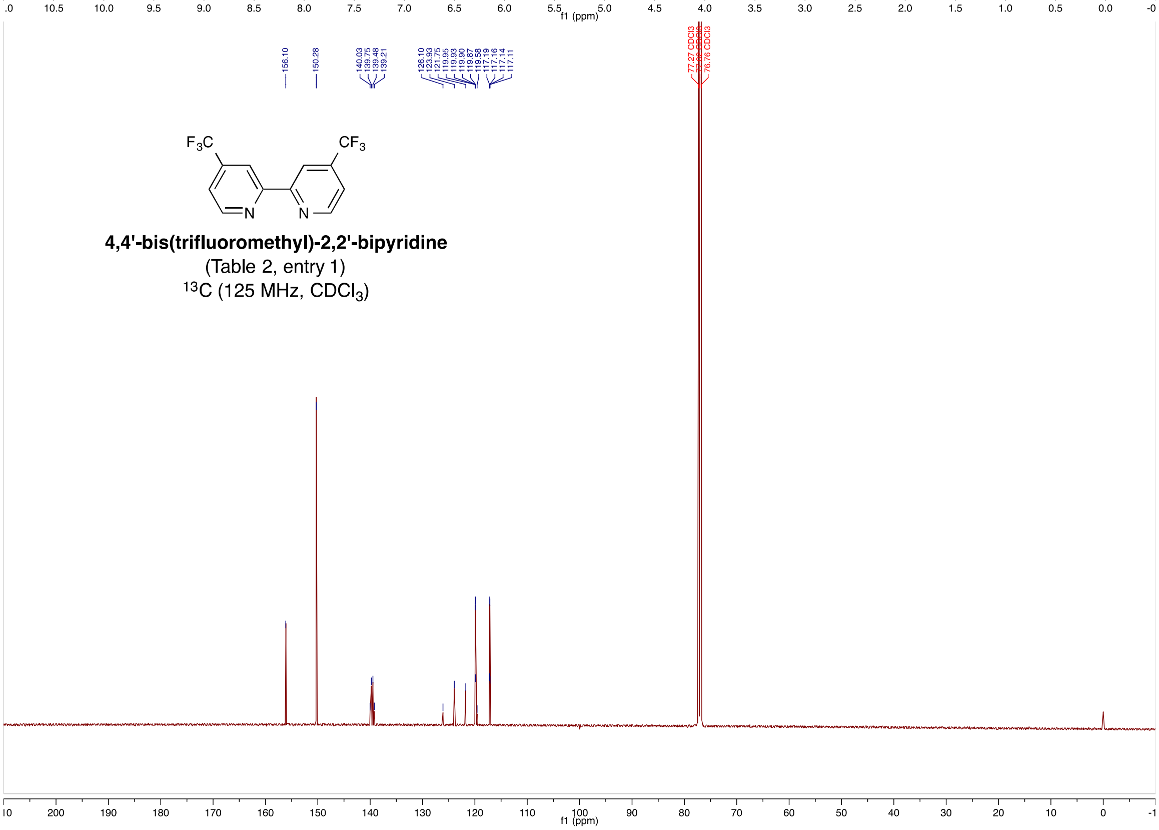
156.10
150.28
140.03
139.75
139.48
139.21
126.10
123.93
121.75
119.95
119.93
119.90
119.87
119.58
117.19
117.16
117.14
117.11
77.27 CDCl3
76.76 CDCl3
F3C
CF3
N
N
4,4'-bis(trifluoromethyl)-2,2'-bipyridine
(Table 2, entry 1)
13C (125 MHz, CDCl3)
f1 (ppm)

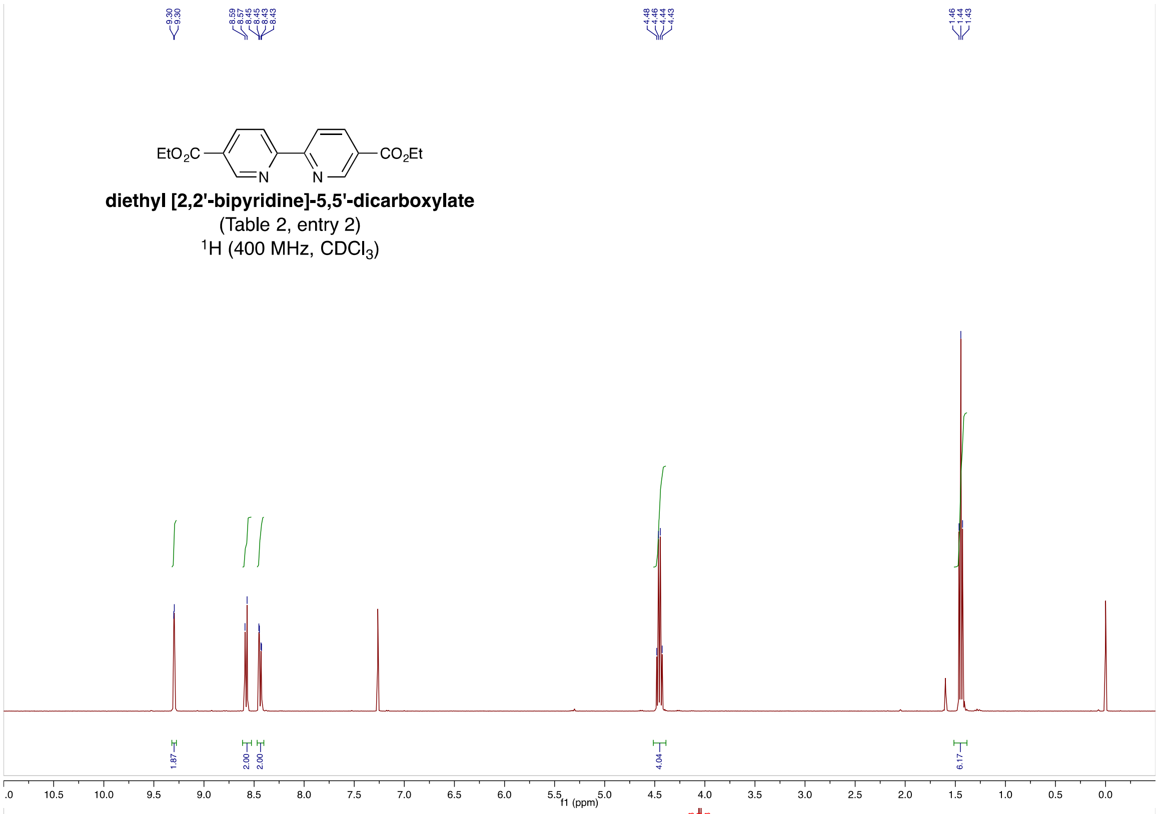
9.30
9.30
8.59
8.57
8.45
8.45
8.43
8.43
4.48
4.46
4.44
4.43
1.46
1.44
1.43
EtO2C
CO2Et
N
N
diethyl [2,2'-bipyridine]-5,5'-dicarboxylate
(Table 2, entry 2)
1H (400 MHz, CDCl3)
1.87
2.00
2.00
4.04
6.17
f1 (ppm)

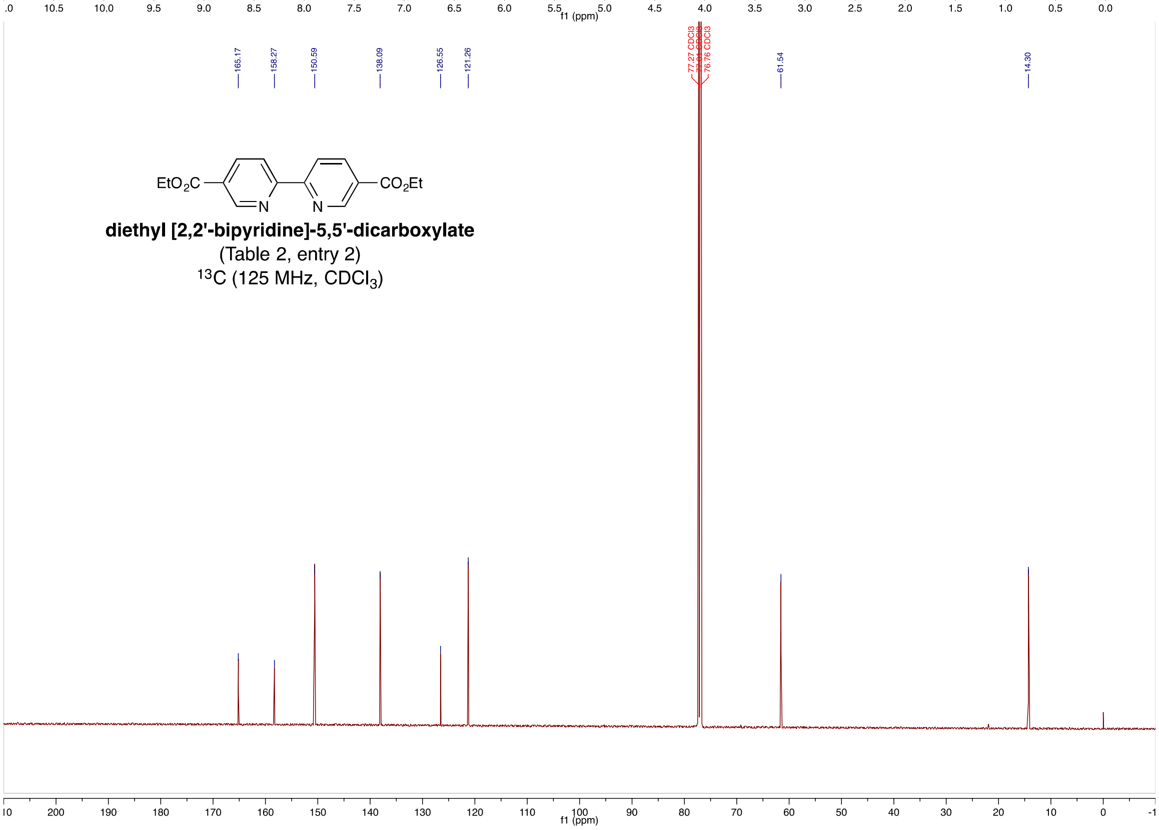
165.17
158.27
150.59
138.09
126.55
121.26
77.27 CDCl3
76.76 CDCl3
61.54
14.30
EtO2C
CO2Et
N
N
diethyl [2,2'-bipyridine]-5,5'-dicarboxylate
(Table 2, entry 2)
13C (125 MHz, CDCl3)
f1 (ppm)

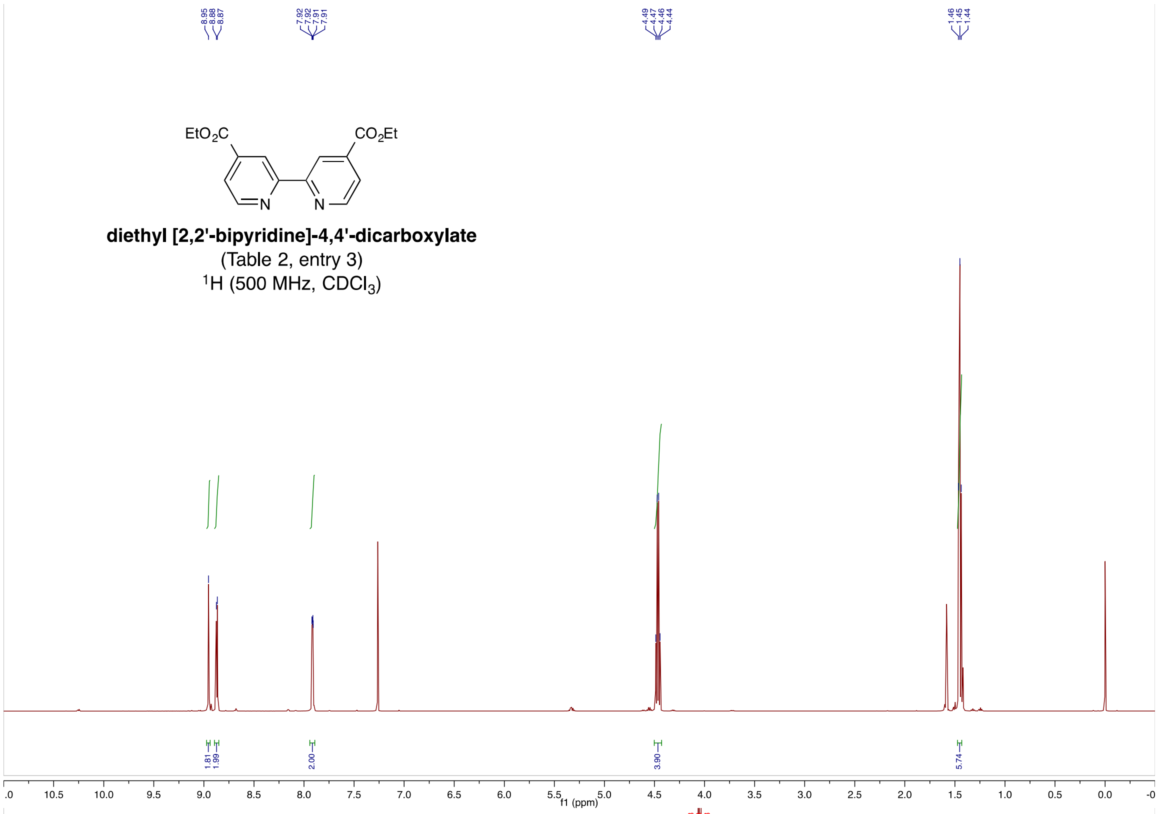
8.95
8.88
8.87
7.92
7.92
7.91
7.91
4.49
4.47
4.46
4.44
1.46
1.45
1.44
EtO2C
CO2Et
N
N
diethyl [2,2'-bipyridine]-4,4'-dicarboxylate
(Table 2, entry 3)
1H (500 MHz, CDCl3)
1.81
1.99
2.00
3.90
5.74
f1 (ppm)

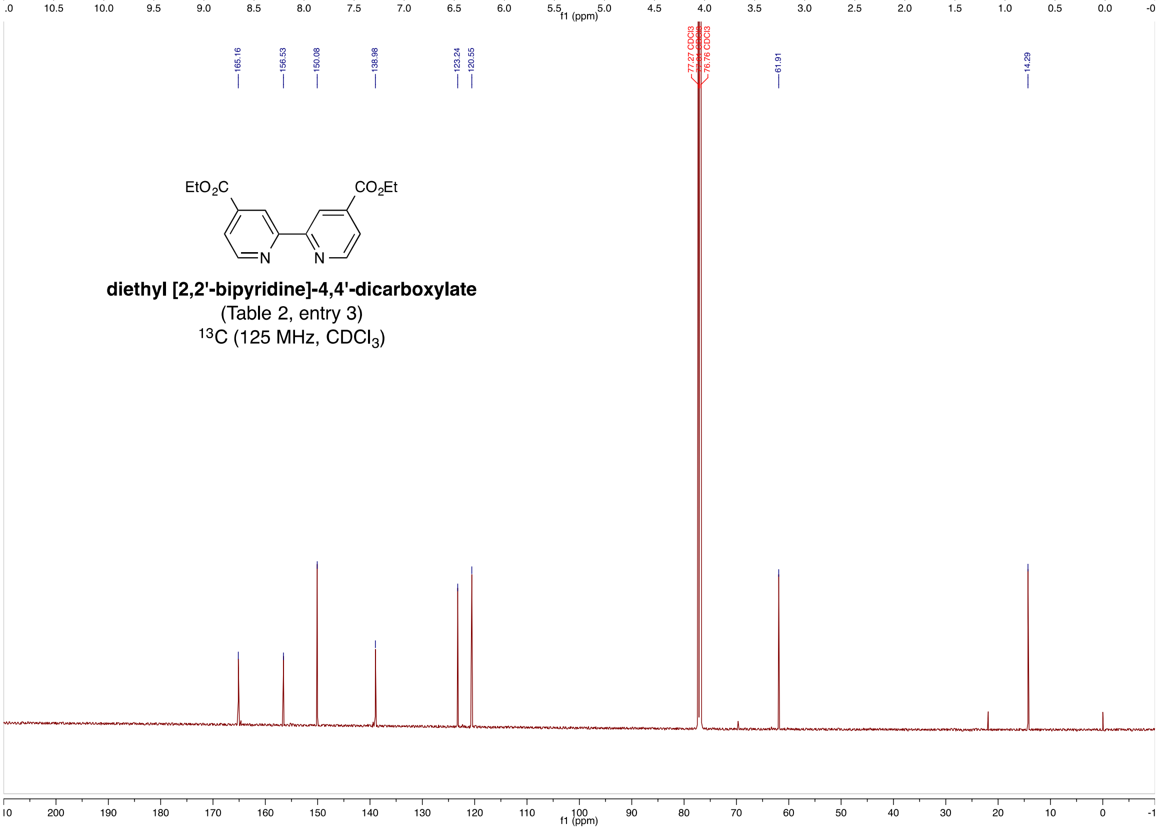
165.16
156.53
150.08
138.98
123.24
120.55
77.27 CDCl3
76.76 CDCl3
61.91
14.29
EtO2C
CO2Et
N
N
diethyl [2,2'-bipyridine]-4,4'-dicarboxylate
(Table 2, entry 3)
13C (125 MHz, CDCl3)
f1 (ppm)

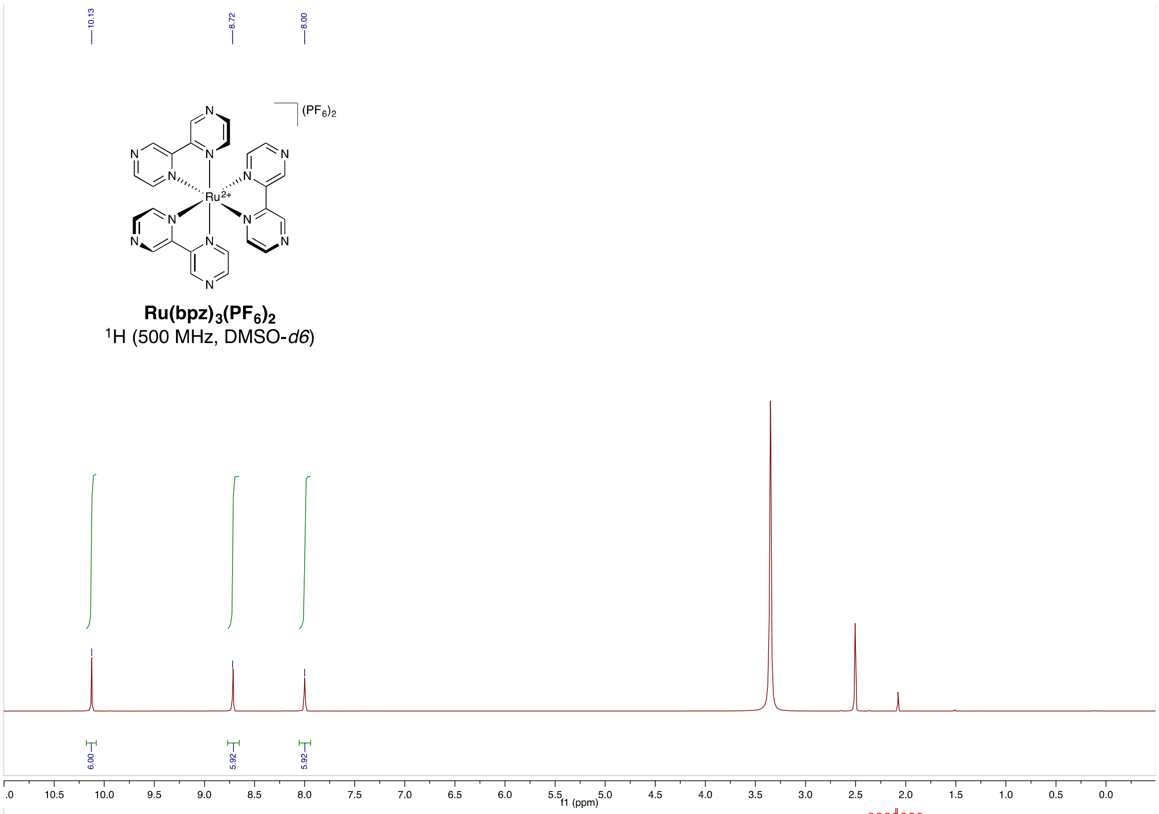

10.13
8.72
8.00
(PF6)2
Ru(bpz)3(PF6)2
1H (500 MHz, DMSO-d6)
6.00
5.92
5.92
f1 (ppm)

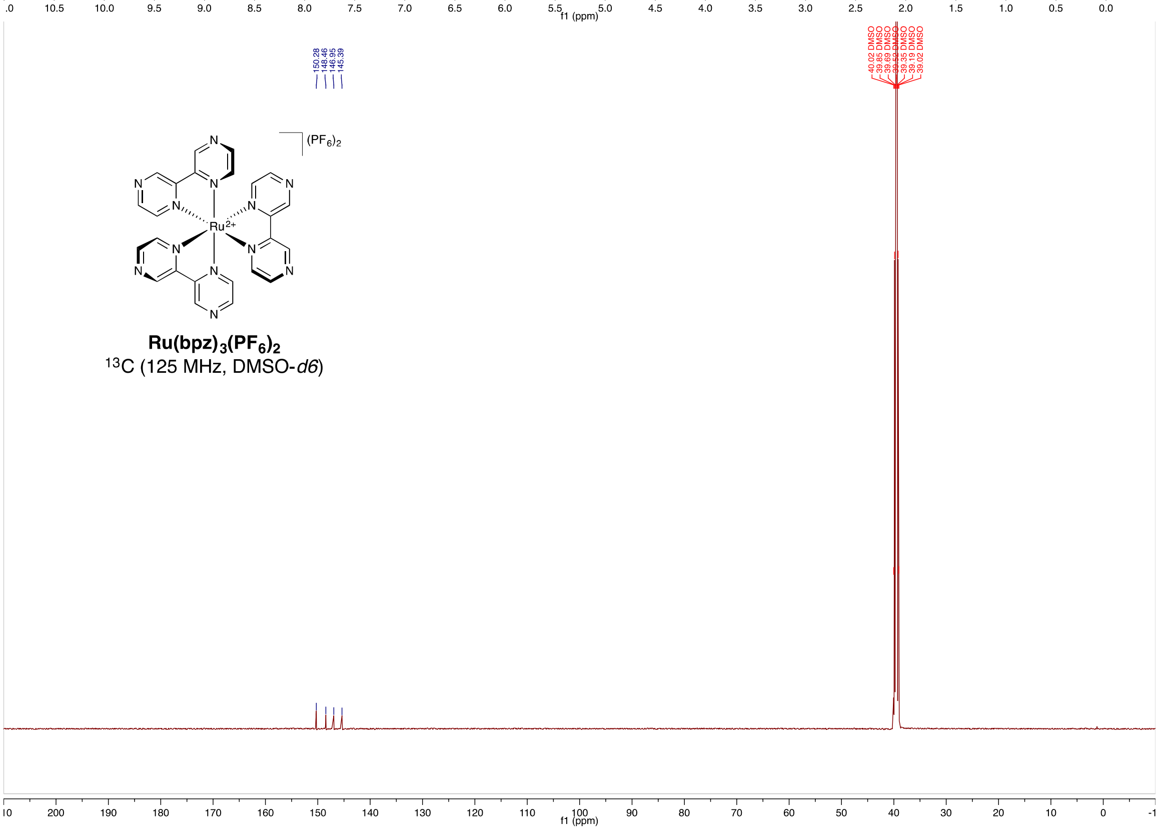

150.28
148.46
146.95
145.39
40.02 DMSO
39.85 DMSO
39.69 DMSO
39.35 DMSO
39.19 DMSO
39.02 DMSO
(PF6)2
Ru(bpz)3(PF6)2
13C (125 MHz, DMSO-d6)
f1 (ppm)

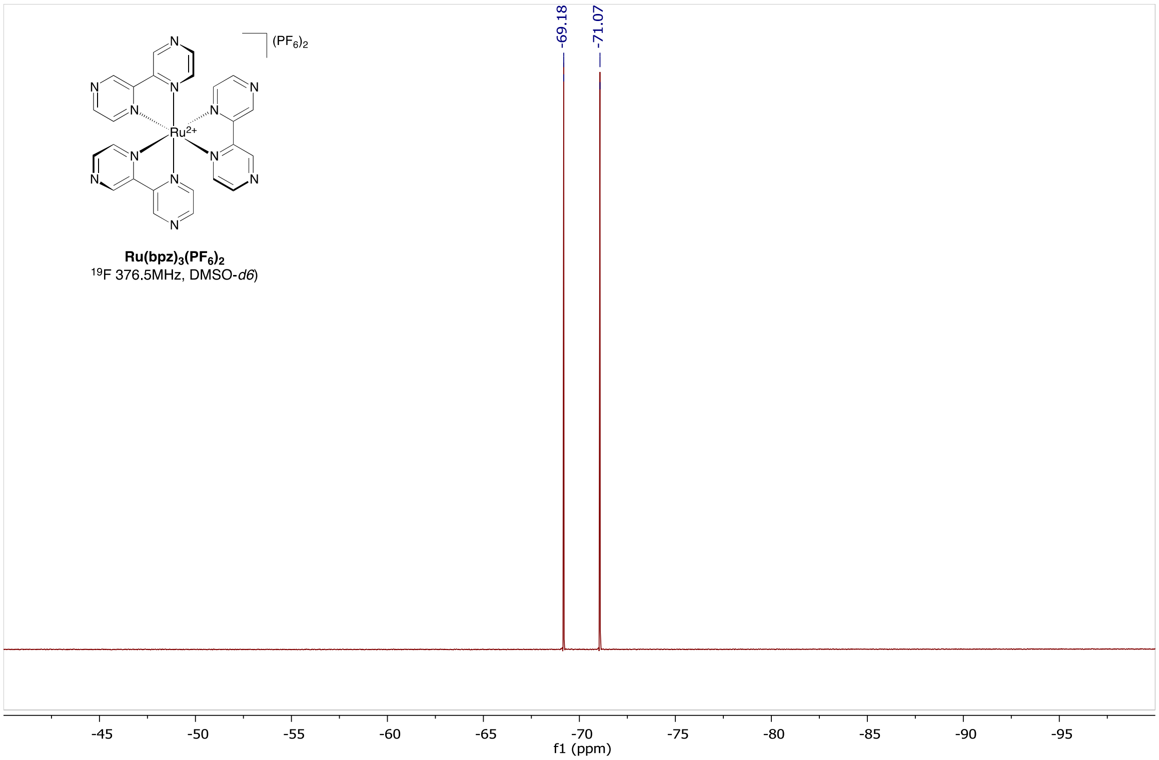

References

[1] Fort, Y.; Becker, S.; Caubère, P. *Tetrahedron* **1994**, *50*, 11893.

[2] Hsu, C.-M.; Li, C.-B.; Sun, C.-H. *J. Chin. Chem. Soc.* **2009**, *56*, 873.

[3] Uhlich, N. A.; Sommer, P.; Bühr, C.; Schürch, S.; Reymond, J.–L.; Darbre, T. *Chem. Commun.* **2009**, 6237.

[4] Ardo, S.; Sun, Y.; Staniszewski, A.; Castellano, F. N.; Meyer, G. J. *J. Am. Chem. Soc.* **2010**, *132*, 6696.

[5] Rillema, D. P.; Allen, G.; Meyer, T. J.; Conrad, D. *Inorg. Chem.* **1983**, *22*, 1617.

[6] Crutchley, R. J.; Lever, A. B. P. *Inorg. Chem.* **1982**, *21*, 2276.
